# The complete mitochondrial genome sequence of *Aulacophora indica*

**DOI:** 10.1080/23802359.2019.1698378

**Published:** 2019-12-12

**Authors:** Weiling Jiang, Xiaolei Yu, Huanyu Zhang, Yu Wang, Xiaoxuan Tian

**Affiliations:** Tianjin State Key Laboratory of Modern Chinese Medicine, Tianjin University of Traditional Chinese Medicine, Tianjin, China

**Keywords:** *Aulacophora indica*, mitochondrial genome

## Abstract

*Aulacophora indica* has been intensively studied since it can cause serious damage to cucurbits. Here, we reported the complete mitochondrial genome of *A. indica* for the first time. The mitochondrial genome is 15,730 bp in length and contained 13 protein-coding genes (PCGs), 2 rRNAs, 22 tRNAs, and an AT-rich control region. Phylogenetic tree indicated that *A. indica* is more closely related to *Aulacophora lewisii* than other Galerucinae beetles.

*Aulacophora indica*, belonging to the subfamily Galerucinae, is distributed all over the world but mostly in the tropics. Adults mostly feed on foliage (parenchyma of the lower leaf surface) producing irregular holes (Ahmad et al. 2013). Therefore, it is a severe pest of commercial crops and causes a serious decline in the yield and quality of crops, especially pumpkins and gourds (Lee and Beenen 2015). Apart from host records and taxonomy, studies on biogenetics of *A. indica* are limited. In this study, the complete mitochondrial genome of *A. indica* was sequenced with the aim to provide basic mitogenome information for future phylogenetic studies of Galerucinae beetles.

Adult specimens of *A. indica* were collected from Nanchang City, Jiangxi Province (115.85°E, 28.68°N), China. The sample was deposited in Tianjin State Key Laboratory of Modern Chinese Medicine (voucher number: ARP-1). Total DNA of *A. indica* was extracted using a Genomic DNA Extraction Kit, following the manufacturer’s instruction. Raw data was generated using the IonTorrent PGM platform (Research and Development Center of Traditional Chinese Medicine, Tianjin, China), and de novo assembly was conducted using MITObim v1.6 (Hahn et al. 2013) with the model of quick flag. MITOS web server (Bernt et al. 2013) was used to annotate genome and tRNAscan-SE (Lowe and Chan 2016) search server was used to confirm tRNA genes.

The length of *A. indica* mitochondrial genome (Genbank accession number: MN686023) is 15,730 bp, including 13 protein-coding genes (PCGs), 2 rRNAs, 22 tRNAs, and an AT-rich control region. The whole genome base composition is 39.5% A, 43.0% T, 11.4% C and 6.1% G. The putative control region is located between tRNA-Ile and 12S rRNA, with the length of 1130 bp long and a total A + T content of 82.5%. Among 13 PCGs, *ND5* (1705 bp) is the longest one and *ATP8* (156 bp) is the shortest one. These genomic features of *A. indica* are similar to the mitochondrial genome of the same genus species *A. lewisii* (Song et al. 2018). Twelve PCGs start with a typic ATN codon, including 6 start with ATT and 6 start with ATG, and only *ND1* starts with TTG. Four PCGs (*COXI*, *COIII*, *ND5*, and *ND4*) are terminated with an incomplete stop codon (TA or T).

To investigate the phylogenetic position of *A. indica*, 15 complete mitochondrial genomes of subfamily Galerucinae and one of subfamily Bruchinae as an outgroup were downloaded from NCBI. A maximum-likelihood tree was constructed using IQ-TREE (Nguyen et al. 2015) under the GTR + F + I + G4 model with 1000 bootstrap replicates. As expected, the phylogenetic analysis clearly indicated that *A. indica* had a close relationship with *Aulacophora lewisii*, and further confirmed that *A. indica* belonged to the tribe Luperini of subfamily Galerucinae (Figure 1).

**Figure 1.**
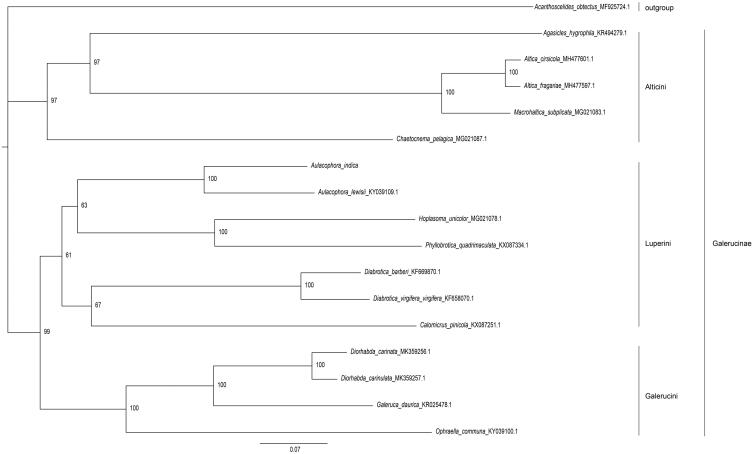
Maximum-likelihood tree inferred from complete mitochondrial genome sequences. Branch support values are presented near each node. GenBank accession numbers follows the species name. Tribe and subfamily of species taxonomy are shown on the right.
